# Association of Hepatitis C and B Virus Infection with CKD and Impact of Hepatitis C Treatment on CKD

**DOI:** 10.1038/s41598-018-36437-6

**Published:** 2019-02-13

**Authors:** Hui Zhang, Hongqin Xu, Ruihong Wu, Ge Yu, Haibo Sun, Juan Lv, Xiaomei Wang, Xiumei Chi, Xiuzhu Gao, Fei Kong, Mingyuan Zhang, Lei Hang, Jing Jiang, Yu Pan, Junqi Niu

**Affiliations:** 1 Department of Hepatology, The First Hospital of Jilin University, Jilin University, Changchun, 130021 China; 2 Department of Hepatology, Jiangsu Taizhou People’s Hospital, Taizhou, 225300 China; 3 Jilin Province Key Laboratory of Infectious Diseases, Laboratory of Molecular Virology, Changchun, 130021 China; 4 Department of Clinical Epidemiology, The First Hospital, Jilin University, No. 71 Xinmin Street, Changchun, 130021 China

## Abstract

Hepatitis C virus (HCV) infection greatly increases the risk of nephropathy. In this observational study, we aimed to explore the relationship between viral hepatitis infection and chronic kidney disease (CKD), identify risk factors, and determine the effect of antiviral treatment on CKD in Chinese patients with chronic HCV infection. A total of 2,435 study subjects were enrolled and divided into four groups: the HCV infection, HBV infection, HBV and HCV co-infection, and uninfected control groups. Of these, 207 patients with chronic hepatitis C (CHC) were given standard dual therapy [subcutaneous injection of recombinant interferon (IFN)-α2b and oral ribavirin (RBV)] for 48 weeks. We found that the prevalence of CKD gradually increased with age in all groups and was significantly increased in patients 60 years or older. Multivariate logistic regression analyses showed that persistent HCV infection was significantly associated with CKD [odds ratio (OR), 1.33; 95% confidence interval (CI), 1.06–1.66; *P* = 0.013], whereas there was no significant link between CKD and spontaneous HCV clearance (OR, 1.23; 95% CI, 0.79–1.90; *P* = 0.364), HBV infection (OR, 0.73; 95% CI, 0.44–1.19; *P* = 0.201), or HBV/HCV co-infection (OR, 1.40; 95% CI, 0.81–2.40; *P* = 0.234). Notably, after anti-HCV therapy, the serum creatinine concentration was significantly decreased (76.0, 75.5–79.4 μmol/L) from the pretreatment level (95.0, 93.0–97.2 μmol/L), both in patients who showed an end of treatment virological response (ETVR) and those who did not (*P* < 0.001). Also, in both the ETVR and non-ETVR groups, the percentages of patients with an estimated glomerular filtration rate (eGFR) ≥90 ml/min/1.73 m^2^ increased significantly (*P* < 0.001), whereas the percentages of those with an eGFR <60 ml/min/1.73 m^2^ significantly decreased (*P* < 0.001). In conclusion, persistent HCV infection was independently associated with CKD, and antiviral treatment with IFN plus RBV can improve renal function and reverse CKD in HCV-infected patients.

## Introduction

Chronic kidney disease (CKD), which is characterized primarily by loss of renal function over time, remains a serious health problem worldwide. A recent cross-sectional survey showed that the overall prevalence of CKD in China is as high as 10.8%, and approximately 120 million individuals suffer from CKD nationwide^1^. In the United States, Europe, Australia, and Japan, the incidence of CKD ranges from 6–11%^2,3^. It has been demonstrated that CKD is more prevalent among patients infected with hepatitis C virus (HCV) than among the general population^4^. In fact, chronic HCV infection can raise the risk of developing CKD by 23%^5^. Two large cohort analyses including more than 150,000 US veterans with chronic HCV infection suggested that this population has a nearly 2-fold greater risk of developing end-stage renal disease (ESRD)^6,7^. From a number of recent independent studies, an updated meta-analysis demonstrated a significant increase in the risk of CKD among HCV-infected patients in comparison with uninfected individuals^8,9^.

The presence of HCV also is associated with rapid deterioration of renal function, suggesting that it is necessary to develop treatments to prevent HCV-induced CKD^10^. In a recent study in a US population, Park and colleagues assessed the risk of CKD development among those with HCV infection as well as the effects of various antiviral treatments on the incidence of CKD in HCV-infected patients^11^. Notably, effective HCV treatment significantly reduced the prevalence of CKD in patients with chronic hepatitis C (CHC)^11^. Similar findings were also demonstrated in several prospective studies, indicating that anti-HCV treatment reduces the risk of developing CKD^12,13^. However, whether antiviral treatment in hepatitis can also support an improvement in renal function and reversal of CKD development requires further assessment. A meta-analysis of 11 clinical trials conducted in Western countries and Japan showed that IFN-α–based antiviral therapy led to a significant decrease in proteinuria and stabilization of serum creatinine levels with greater improvement in protein excretion in CHC patients^14^. The heterogeneity of the demographic data, nature and stage of kidney disease, as well as the severity of liver injury and extrahepatic manifestations may lead to different results, and it remains unknown whether IFN-based dual therapy with IFN plus RBV, the main components of HCV treatment in mainland China due to the high cost of direct-acting antiviral agents (DAAs), can improve renal function or reverse CKD in HCV-infected patients in a Chinese population.

Unlike HCV infection, whether HBV infection can increase the risk of CKD development and promote CKD progression has not been appropriately investigated. A meta-analysis found no correlation between HBV sero-positive status and the prevalence of CKD or proteinuria in a cross-sectional survey^15^.

In the present study, we aimed to investigate the association between HBV infection, HCV infection, or HBV/HCV co-infection and CKD as well as to analyze the effect of anti-HCV therapy with IFN-based dual therapy on CKD recovery. Fuyu is an endemic area for HCV infection and provides an excellent setting for this type of study, as it is suitable for examining the relationship of IFN-based therapy for HCV with CKD due to the high prevalence of both HCV infection and anti-viral treatment^16,17^. The results gained through this study may provide critical information that will assist in the development of early intervention measures for preventing kidney damage caused by pathogenic hepatic viruses.

## Results

### Demographic and clinical characteristics of the study participants

A total of 2,435 subjects completed the survey and examination in this study. The mean age was 50.3 ± 10.3 years, and 52.5% of patients were women. The study subjects were divided into four groups based on the types of viral hepatitis, according to the following distribution: HCV infection (38.3%), HBV infection (4.8%), HBV/HCV co-infection (2.9%), and uninfected controls (54.0%). The demographic and clinical characteristics of the study subjects in the four groups are summarized in Table 1. The HCV infection group had an older mean age (54.6 ± 8.7 years), higher rates of diabetes mellitus (10.8%) and CKD (32.1%), and higher levels of hemoglobin [152 (140–163) g/L], alanine aminotransferase (ALT) [38 (23–72) U/L], total bilirubin [13.8 (9.9–18.5) μmol/L], serum urea nitrogen (SUN) [5.6 (4.6–6.7) mmol/L], and serum creatinine (Scr) [67.0 (58.0–76.4) μmol/L] compared to the HBV group, which had a mean age of 46.0 ± 10.2 years and CKD prevalence of 19.7%. Notably, the uninfected control group had higher levels of platelets [238 (198–275) × 10^9^/L], albumin [46.9 (44.8–49.9) g/L], triglycerides [1.7 (1.1–2.8) mmol/L], and cholesterol [5.0 (4.4–5.8) mmol/L] compared with the HCV infection, HBV infection, and HBV/HCV co-infection groups.

**Table 1:** Baseline characteristics of chronic hepatitis patients and controls.

	Infection status				*P*	Significantly different
	^a^HCV alone	^b^HBV alone	^c^Co-infection	^d^Controls		
Sample	932(38.3)	117(4.8)	70(2.9)	1316(54.0)		
Age (y)	54.6 ± 8.7	46.0 ± 10.2	52.0 ± 7.0	47.6 ± 10.5	<0.001	ad cd ab ac bc
Sex						
Men	560(60.1)	54(46.2)	42(60.0)	501(38.1)	<0.001	ad cd ab
Women	372(39.9)	63(53.8)	28(40.0)	815(61.9)		
Smoking	586(62.9)	43(36.8)	47(67.1)	479(36.4)	<0.001	ad cd ab bc
Sharing syringes	416(44.6)	15(12.8)	26(37.1)	156(11.9)	<0.001	ad cd ab bc
Diabetes mellitus	101(10.8)	6(5.1)	6(8.6)	84(6.4)	0.001	ad
BMI (kg/m^2^)	24.0 ± 3.6	24.5 ± 3.5	24.1 ± 3.6	24.4 ± 3.5	0.051	
Hemoglobin (g/L)	152(140,163)	149(136,162)	150(141,158)	146(137,159)	<0.001	ad
Platelet count (×10^9^/L)	185(146,222)	204(167,244)	188(134,231)	238(198,275)	<0.001	ad bd cd ab
Albumin (g/L)	44.9(42.6,47.7)	46.3(43.8,49.4)	44.9(42.6,46.9)	46.9(44.8,49.9)	<0.001	ad bd cd ab bc
Alanine aminotransferase (U/L)	38(23,72)	24(17,38)	36(20,58)	19(14,29)	<0.001	ad bd cd ab bc
Total bilirubin (μmol/L)	13.8(9.9,18.5)	13.3(9.4,18.6)	12.7(9.3,18.0)	11.3(8.1,15.6)	<0.001	ad bd cd
Triglyceride(mmol/L)	1.3(0.9,2.0)	1.3(0.9,2.0)	1.3(0.9,2.2)	1.7(1.1,2.8)	<0.001	ad bd cd
Cholesterol (mmol/L)	4.5(4.0,5.2)	4.7(4.2,5.4)	4.7(4.1,5.4)	5.0(4.4,5.8)	<0.001	ad bd cd
Fasting plasma glucose (mmol/L)	4.2(3.6,5.0)	4.1(3.6,4.5)	4.1(3.7,5.1)	4.2(3.7,4.8)	0.175	
Serum urea nitrogen (mmol/L)	5.6(4.6,6.7)	5.1(4.2,6.3)	5.4(4.4,6.6)	5.1(4.3,6.3)	<0.001	ad ab
Serum creatinine (mol/L)	67.0(58.0,76.4)	63.6(55.6,73.0)	64.7(55.3,76.3)	63.0(54.7,74.2)	<0.001	ad ab
Urinary albumin to creatinine ratio (mg/mmol)					0.47	
<3	681(73.1)	94(80.3)	54(77.1)	978(74.3)		
3–29	233(25.0)	21(17.9)	15(21.4)	323(24.5)		
≥30	18(1.9)	2(1.7)	1(1.4)	15(1.1)		
^*^Albuminuria	255(26.9)	23(19.6)	17(22.8)	343(25.6)	0.336	
eGFR					<0.001	ad ab
≥90	548(58.8)	88(75.2)	48(68.6)	920(69.9)		
60–89	318(34.1)	28(23.9)	16(22.9)	355(27.0)		
<60	66(7.1)	1(0.9)	6(8.6)	41(3.1)		
CKD, stages					<0.001	
1	148(15.9)	16(13.7)	10(14.3)	238(18.1)		
2	85(9.1)	6(5.1)	5(7.1)	85(6.5)		
≥3	66(7.1)	1(0.9)	6(8.6)	41(3.1)		
Total	299(32.1)	23(19.7)	21(30.0)	364(27.7)	0.015	

### Multivariate analysis of variables associated with CKD

Age, gender, cigarette smoking, viral hepatitis infection, diabetes mellitus, body mass index (BMI), hemoglobin, albumin, total bilirubin (TBIL), triglycerides, and cholesterol levels were significantly correlated with CKD, and thus, these variables were selected for subsequent multivariable logistic regression analysis. As shown in Table 2, persistent HCV infection [odds ratio (OR), 1.33; 95% confidence interval (CI), 1.06–1.66] was identified to be a significant independent risk factor for CKD (*P* = 0.013), but not spontaneous HCV clearance [OR, 1.23; 95% CI, 0.79–1.90 (*P* = 0.364)], HBV infection [OR, 0.73; 95% CI, 0.44–1.19 (*P* = 0.201)], or HBV/HCV co-infection [OR, 1.40; 95% CI, 0.81–2.40 (*P* = 0.234)]. Other independent risk factors significantly associated with CKD included age, female gender, diabetes mellitus, and levels of TBIL, triglycerides, and cholesterol (Table 2).

**Table 2:** Univariate and multivariate regression analyses of risk factors associated with CKD.

Variables	Univariate (95% CI)	*P*	Effect *P*	Multivariate (95% CI)	*P*	Effect *P*
Age (y)			<0.001			<0.001
≤40	1.00(ref.)			1.00(ref.)		
41–50	0.93(0.70–1.24)	0.630		0.84(0.63–1.11)	0.208	
51–60	1.1(0.83–1.46)	0.520		0.93(0.69–1.25)	0.615	
61–70	2.09(1.53–2.87)	<0.001		1.82(1.29–2.56)	0.001	
≥71	6.31(3.57–11.16)	<0.001		5.47(3.04–9.82)	<0.001	
Sex						
Female	1.00(ref.)			1.00(ref.)		
Male	0.76(0.64–0.91)	0.002		0.80(0.66–0.98)	0.031	
Smoking						
No	1.00(ref.)					
Yes	1.10(1.00–1.40)	0.162				
Viral hepatitis infection status			<0.001			0.044
Controls	1.00(ref.)			1.00(ref.)		
Spontaneous HCV clearance	1.17(0.77, 1.78)	0.465		1.23(0.79–1.90)	0.364	
HCV persistence alone	1.24(1.03–1.50)	0.024		1.33(1.06–1.66)	0.013	
HBV alone	0.64(0.40–1.03)	0.060		0.73(0.44–1.19)	0.201	
Co-infection	1.12(0.66–1.90)	0.670		1.40(0.81–2.40)	0.234	
Diabetes mellitus						
No	1.00(reference)			1.00(reference)		
Yes	2.42(1.80–3.25)	<0.001		2.10(1.54–2.88)	<0.001	
BMI (kg/m^2^)	0.998(0.974–1.023)	0.877				
Hemoglobin (g/L)	0.996(0.991–1.001)	0.128				
Albumin (g/L)	0.990(0.960–1.010)	0.314				
Total bilirubin (μmol/L)	0.97(0.96–0.98)	<0.001		0.98(0.96–0.99)	0.001	
Triglycerides (mmol/L)	1.09(1.04–1.14)	<0.001		1.06(1.01–1.12)	0.021	
Cholesterol (mmol/L)	1.19(1.09–1.29)	<0.001		1.11(1.01–1.22)	0.030	

### Effect of antiviral therapy on renal function in CHC patients

**Figure 1:**
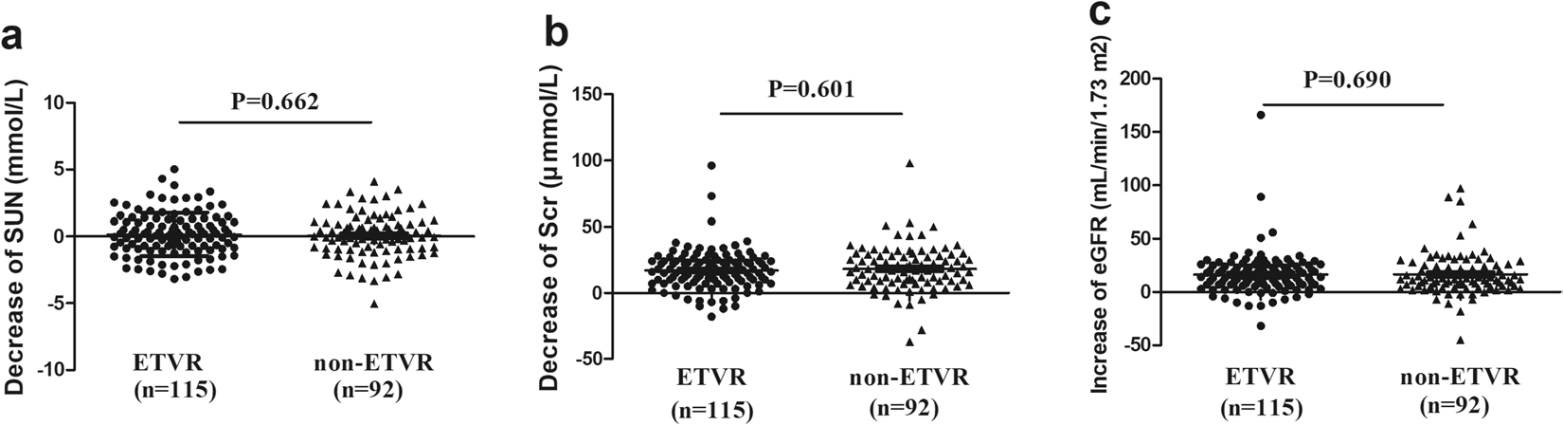
The changes in serum urea nitrogen, serum creatinine, and eGFR before and after antiviral therapy in the ETVR versus non-ETVR groups. (**a**) Decrease in serum urea nitrogen (SUN); (**b**) decrease in serum creatinine (Scr); and (**c**) increase in eGFR.

Overall, 207 CHC patients received standard dual therapy (subcutaneous injection of recombinant IFN-α2b plus oral RBV) for 48 weeks. Upon completion of anti-HCV therapy, 115 (55.6%) patients showed an end of treatment virological response (ETVR), whereas 92 (44.4%) patients did not exhibit an ETVR in our study (not-ETVR). In the total cohort, significant changes in plasma creatinine and estimated glomerular filtration rate (eGFR) were seen after the end of the anti-viral therapy in both the ETVR and non-ETVR groups (Table 3). Furthermore, the levels of serum creatinine in the two groups following the antiviral therapy were 75.0 (70.0–85.0) μmol/L and 77 (67.2–86.8) μmol/L, respectively, which were significantly lower than the pretreatment levels of 95.0 (83.0–103.3) μmol/L and 95.0 (85.3–105.8) μmol/L, respectively (*P* < 0.001). Additionally, the eGFR was improved after the end of the antiviral treatment; the proportion of patients with an eGFR ≥90 ml/min/1.73 m^2^ was significantly increased (*P* < 0.001), whereas that of individuals with an eGFR <60 ml/min/1.73 m^2^ was decreased significantly in both the ETVR and non-ETVR groups (*P* < 0.001). Notably, the antiviral treatment led to a significant reduction in the prevalence of CKD (*P* < 0.001), whereas it did not alter the incidence of albuminuria. We also compared the changes in serum urea nitrogen, serum creatinine, and eGFR before and after antiviral therapy in the ETVR versus non-ETVR groups, and no significant differences between the groups were found. As shown in Fig. 1, the serum urea nitrogen decreased by 0.16 ± 1.62 mmol/L in the ETVR group and by 0.06 ± 1.63 mmol/L in the non-ETVR group (*P* = 0.662); the serum creatinine decreased by 17.10 ± 15.20 mmol/L in the ETVR group and by 18.28 ± 17.37 mmol/L in the non-ETVR group (*P* = 0.601); and the eGFR increased by 16.45 ± 20.36 mmol/L in the ETVR group and by 16.58 ± 20.08 mmol/L in the non-ETVR group (*P* = 0.690).

**Table 3:** Comparison of indicators of kidney disease before and after antiviral therapy.

	ETVR (n = 115)		*P* ^*^	non-ETVR (n = 92)		*P* ^#^
	Before	After		Before	After	
Serum urea nitrogen (mmol/L)	5.7(4.8–6.6)	5.6(4.7–6.6)	0.388	5.7(4.8–6.5)	5.7(4.8–6.4)	0.664
Serum creatinine (µmol/L)	95.0(83.0–103.0)	75.0(70.0–85.0)	<0.001	95.0(85.3–105.8)	77.0(67.2–86.8)	<0.001
Albumin to creatinine ratio (mg/mmol)			0.647			0.493
<3	85(73.9)	90(78.30)		67(72.8)	72(78.3)	
3–29	28(24.3)	24(20.9)		25(27.2)	20(21.7)	
≥30	2(1.7)	1(0.9)		0(0)	0(0.0)	
^*^Albuminuria	30(26.1)	25(21.7)	0.44	25(27.2)	20(21.7)	0.39
eGFR (mL/min/1.73 m^2^)			<0.001			<0.001
≥90	21(18.3)	56(48.7)	—	13(14.1)	35(38.0)	—
60–89	69(60.0)	53(46.1)	<0.001	51(55.4)	48(52.2)	<0.001
<60	25(21.7)	6(5.2)	<0.001	28(30.4)	9(9.8)	<0.001
CKD stage			0.022			0.024
1	10(20.4)	9(30.0)	—	2(4.7)	4(14.8)	—
2	14(28.6)	15(50.0)	0.774	13(30.2)	14(51.9)	0.665
3	25(51.0)	6(20.0)	<0.001	28(65.1)	9(33.3)	0.058
Total	49(12.6)	30(26.1)	0.008	43(52.4)	27(29.3)	0.002

## Discussion

In this study, we investigated the prevalence of CKD in mainland China, a high endemic area of HCV infection, for the first time and determined the risk factors associated with CKD. The major novel findings are summarized as follows. (1) The prevalence of CKD in uninfected controls was 27.7%, which was much higher than that previously reported for the general population of rural areas in northern regions (16.9%)^1^. This may be due to an inherent bias associated with subject selection, as the mean age of all participants in this study was 47.6 years. Therefore, an older age may have influenced the high rate of CKD found in this study. (2) The prevalence of CKD in the HBV infection group was the lowest, which may be associated with the younger age distribution and lower prevalence of diabetes (5.1%). This finding was consistent with a previous study in which the HBV infection group had a lower prevalence of CKD (14.3%) than an HCV infection group (22.1%) and HBV/HCV co-infection group (17.2%)^8^. (3) The HCV infection group had the highest prevalence of diabetes (10.8%), which is consistent with several studies, showing that the prevalence of diabetes mellitus was significantly greater in patients with HCV infection^18–20^. Although the underlying mechanisms need to be elucidated, it appears that dysregulation of cytokines, intracellular oxidative stress, suppression of insulin downstream signaling, modulation of incretins, and pancreatic β-cell dysfunction are contributing factors^21–23^.In the present study, multivariate logistic regression analysis revealed that age >60 years, female gender, diabetes, lower TBIL, higher triglycerides, and higher total cholesterol were significant independent risk factors associated with the development of CKD, and these findings were consistent with other studies^24,25^. In this study, we also found that higher levels of TBIL were significantly associated with CKD (OR, 0.98; 95% CI, 0.96–0.99; *P* < 0.001). Mashitani and colleagues found that the serum bilirubin levels were associated with diabetic nephropathy progression in type 2 diabetes patients^26^. As bilirubin is a normal metabolite of human hemoglobin, and a number of recent studies have shown that bilirubin has a dose-dependent antioxidant effect, bilirubin production potentially may act as a compensatory defense mechanism when renal function is mildly reduced^27^.

After having adjusted for gender, age, and the other potential confounders, persistent HCV infection was identified as an independent risk factor for CKD in our study. A study of male veterans in the US demonstrated that HCV infection was significantly associated with membrano proliferative glomerulo nephritis (MPGN)^28^. Another Taiwanese study found that HCV infection was significantly related to the high prevalence and severity of CKD^27^. Furthermore, the incidence of ESRD was reported to be 2.14-fold greater in patients with chronic HCV infection than in those without HCV infection^29^. As CKD is highly prevalent in hepatitis C patients, all of the above findings indicate that HCV infection is an independent risk factor for CKD. Given the aforementioned potential renal involvement, annual screening for CKD in patients with CHC is highly recommended. Our results demonstrate that HCV/HBV co-infection was not associated with CKD, which may due to replication inhibition of HBV and HCV in these patients^30^. This conjecture is also in good agreement with the possibility that HCV-related kidney diseases may result from HCV directly and immune-mediated kidney damage^31^.

In the anti-viral treatment cohort, we found that the antiviral therapy consisting of IFNα-2b combined with RBV decreased the serum creatinine level and improved both the eGFR and severity of CKD, which is in agreement with other studies in western countries and Japan^14^. However, the repair of kidney damage following antiviral therapy was not linked to HCV RNA clearance. Compared with the non-ETVR group, significant decreases in serum creatinine and serum urea nitrogen along with an improvement in eGFR were not observed in the ETVR group in our study. In fact, when we divided the patients into ETVR and non-ETVR groups, the number of cases (115 patients with ETVR and 92 patients without ETVR achieved) in our study was too small for statistical analysis. Therefore, a limitation is the small number of study subjects making it difficult to perform subgroup analysis in our treatment cohort.

This study has several other limitations. First, the dual therapy with pegylated (PEG)-IFN plus RBV was previously used to treat chronic HCV infection. However, a number of limitations have been well recognized, which include low sustained virologic response (SVR) rates (approximately 50% of SVRs for genotype I), numerous adverse effects associated with the dual therapy, and the requirement of a long treatment duration of the dual therapy (24 or 48 weeks). The treatment of HCV infection has been changing rapidly, and newly developed DAAs have shown greater clinical efficacy than the dual therapy in patients with chronic HCV infection despite their high cost. Therefore, the effects of DAAs on renal function need to be studied in the future. Second, we enrolled 117 patients with HBV infection and 70 individuals with HBV/HCV co-infection, and thus, the sample sizes of these groups were relatively small for evaluating the prevalence of CKD. Third, the status of CKD was determined at the baseline cross-sectional screening survey, while data on the duration of renal damage and decline of GFR in the study subjects were not available in the current study. Furthermore, some other risk factors associated with the development of CKD, such as cigarette smoking, cirrhosis, hypertension, angiotensin-converting enzyme inhibitor (ACEi) use, angiotensin receptor blocker (ARB) use, and cardiovascular diseases, were not taken into consideration in our study. Therefore, further investigation is underway in our center to determine if these parameters have any other confounding effects.

In conclusion, this study has provided important scientific evidence that persistent HCV infection is strongly associated with CKD. The findings have also demonstrated that anti-viral therapy in patients with hepatitis C can improve or restore kidney function in our study.

## Methods

### Study subjects

This observational study was conducted in Fuyu city (Jilin, China), an area with a prevalence of HCV infection as high as 40%. A total of 3,219 participants completed the cross-sectional survey and physical examination in September 2012 at the First Hospital of Jilin University. After excluding 784 participants with no serum creatinine measurement or urine protein results, the remaining 2,435 individuals were included in the final analysis. These patients included 932 with HCV infection, 117 with HBV infection, 70 with HBV/HCV coinfection, and 1,316 uninfected healthy controls. HCV infection was defined by positivity for HCV antibody, HBV infection by positivity for HBsAg, and co-infection with HBV and HCV by positivity for HBsAg and HCV antibody. Uninfected healthy controls had neither HBV infection nor HCV infection. The HCV infection group was further divided into two sub-groups: 110 patients with spontaneously cleared HCV (positive for HCV antibody in the absence of detectable HCV-RNA) and 822 patients with HCV persistence (anti-HCV positive/HCV-RNA positive).

Moreover, 207 CHC patients from the above cohort were enrolled in a subsequent cohort of patients who receive anti-HCV treatment consisting of standard dual therapy with subcutaneous injection of recombinant IFN-α2b (5 MU, three times/week; BeiJing Kavin Technology Share-holding Co, Ltd., Beijing, China) in combination with oral RBV (800 mg/day for <60 kg body weight, 1000 mg/day for 60–75 kg, and 1200 mg/day for >75 kg) for 48 weeks. During the treatment period, the 207 CHC patients were scheduled for assessment of both the efficacy and safety of the dual therapy at different time points, including baseline and 4, 12, 24, and 48 weeks on the standard dual therapy.

Written informed consent was obtained from every participant in this study, and the study protocol was reviewed and approved by the Ethics Committee of the First Hospital of Jilin University.

### Laboratory tests

Blood samples were taken in the morning after more than 8 h of fasting, and the laboratory tests for liver function, renal function, blood glucose, fasting insulin, and C peptide were performed using a Synchron LX®20 autoanalyser (Beckman Coulter, Brea, CA, USA). At the Clinical Laboratory, the First Hospital of Jilin University. Levels of HCV RNA were examined by quantitative real-time polymerase chain reaction (qRT-PCR) using the COBAS AmpliPrep/COBAS TaqMan HCV Test following the manufacturer’s instructions (Roche Molecular Systems, Pleasanton, CA, USA) with the limit of detectability as low as 15 IU/ml. Levels of anti-HCV and HBsAg were determined on an Abbott ARCHITECT i2000SR. HCV genotyping was conducted using multicolor fluorescence PCR with an HCV RNA genotype kit (BioAssay Science & Technology Co. Ltd, Beijing, China).

The following Cockcroft-Gault formula was used to calculate the eGFR: eGFR = [(140-age) × weight (kg)]/[0.818 × SCr (μmol/L)] × 0.85 (if female), in which SCr represents serum creatinine. The stages of CKD were determined by the patient’s level of eGFR and the status of albuminuria according to the criteria of the Kidney Disease Outcomes Quality Initiative (K/DOQI)^32,33^. CKD stage 1 was defined as eGFR ≥ 90 mL/min/1.73 m^2^ in combination with albuminuria; CKD stage 2 was defined as eGFR of 60–89 mL/min/1.73 m^2^ with the presence of albuminuria; and CKD stage 3 or above was defined as eGFR < 60 ml/min/1.73 m^2^. The diagnosis and stages of CKD were assigned at the baseline cross-sectional screening survey in all study participants. Also the diagnosis and stages of CKD were assigned at baseline and at 4, 12, 24, and 48 weeks of the standard dual therapy given to the 207 CHC patients in the anti-HCV treatment cohort.

### Statistical analysis

Statistical analysis was conducted using SPSS software package 18.0 (SPSS Inc., Chicago, IL, USA). Continuous variables are presented as means ± standard deviation (SD) or median (inter quartile), and categorical variables are expressed as frequency (%). Continuous variables were compared among the four groups using analysis of variance (ANOVA) tests, and comparisons between any two groups were performed using the least significant difference (LSD) test. Categorical variables were analyzed using a chi-squared test or the Fisher’s exact test. Univariate and multivariate logistic binary regression analyses were used to identify potential confounding variables. For the analysis of data before and after anti-HCV therapy, we used the Wilcoxon signed-rank test, the McNemar test, or the χ^2^ test. A *P*-value < 0.05 was considered statistically significant.

## Data Availability

The datasets generated and/or analyzed during the current study are available from the corresponding author upon request.
